# The Function of Two Radical‐SAM Enzymes, HcgA and HcgG, in the Biosynthesis of the [Fe]‐Hydrogenase Cofactor

**DOI:** 10.1002/anie.202213239

**Published:** 2022-11-17

**Authors:** Francisco J. Arriaza‐Gallardo, Sebastian Schaupp, Yu‐Cong Zheng, Mohd Farid Abdul‐Halim, Hui‐Jie Pan, Jörg Kahnt, Georgia Angelidou, Nicole Paczia, Xile Hu, Kyle Costa, Seigo Shima

**Affiliations:** ^1^ Max Planck Institute for Terrestrial Microbiology Karl-von-Frisch-Straße 10 35043 Marburg Germany; ^2^ Department of Plant and Microbial Biology University of Minnesota Twin Cities, St. Paul Minnesota USA; ^3^ Laboratory of Inorganic Synthesis and Catalysis Institute of Chemical Sciences and Engineering Ecole Polytechnique Fédérale de Lausanne (EPFL) ISIC-LSCI, BCH 3305 Lausanne 1015 Switzerland

**Keywords:** Acyl Ligands, Biosynthesis, FeGP Cofactor, Radical S-Adenosyl Methionine Enzymes, [Fe]-Hydrogenase

## Abstract

In the biosynthesis of the iron‐guanylylpyridinol (FeGP) cofactor, 6‐carboxymethyl‐5‐methyl‐4‐hydroxy‐2‐pyridinol (**1**) is 3‐methylated to form **2**, then 4‐guanylylated to form **3**, and converted into the full cofactor. HcgA‐G proteins catalyze the biosynthetic reactions. Herein, we report the function of two radical *S*‐adenosyl methionine enzymes, HcgA and HcgG, as uncovered by in vitro complementation experiments and the use of purified enzymes. In vitro biosynthesis using the cell extract from the *Methanococcus maripaludis* Δ*hcgA* strain was complemented with HcgA or precursors **1**, **2** or **3**. The results suggested that HcgA catalyzes the biosynthetic reaction that forms **1**. We demonstrated the formation of **1** by HcgA using the 3 kDa cell extract filtrate as the substrate. Biosynthesis in the Δ*hcgG* system was recovered by HcgG but not by **3**, which indicated that HcgG catalyzes the reactions after the biosynthesis of **3**. The data indicated that HcgG contributes to the formation of CO and completes biosynthesis of the FeGP cofactor.

## Graphical abstract

The FeGP cofactor is the prosthetic group of [Fe]‐hydrogenase, which catalyzes reversible hydride transfer from H_2_ to an organic substrate. HcgA and HcgG are involved in the biosynthesis of the FeGP cofactor. In vitro biosynthesis experiments indicated the functions of these two radical *S*‐adenosyl methionine enzymes. HcgA biosynthesizes the initial pyridinol precursor, and HcgG contributes to the formation of the CO and acyl ligands.

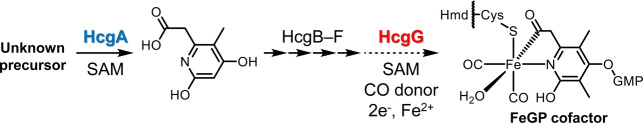

H_2_‐forming methylenetetrahydromethanopterin (methylene‐H_4_MPT) dehydrogenase ([Fe]‐hydrogenase or Hmd) is involved in the hydrogenotrophic methanogenic pathway and catalyzes the reversible hydride transfer from H_2_ to methenyl‐H_4_MPT^+^, which forms methylene‐H_4_MPT. The iron‐guanylylpyridinol (FeGP) cofactor is the prosthetic group of [Fe]‐hydrogenase and contains a low spin Fe^II^ in complex with an acyl ligand from a pyridinol derivative, the pyridinol nitrogen and two CO ligands (Figure 1).^[1]^ The pyridinol derivative contains a guanosine monophosphate substituent, which leads the cofactor to bind at the nucleotide‐binding domain of the protein.^[2]^ In the holoenzyme, a cysteine residue provides a thiolate ligand, which covalently binds the FeGP cofactor to the protein.^[1g]^ The open coordination site *trans* to the acyl ligand is occupied with a water molecule in the resting state and is proposed to be the H_2_ binding site.^[1e]^

**Figure 1:**
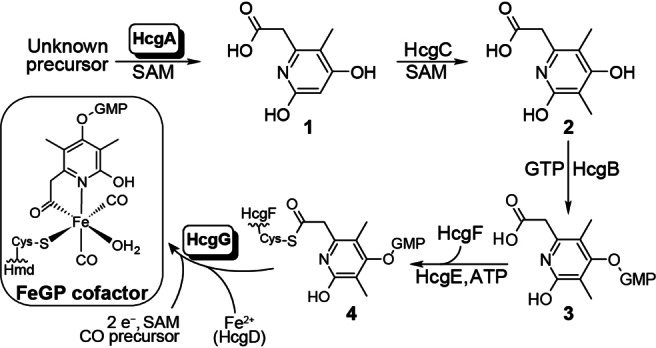
Biosynthetic sequence of the FeGP cofactor catalyzed by HcgA‐G. The catalytic functions of HcgA and HcgG were identified in this work.

The *hmd*‐cooccurring genes (*hcgA*‐*G*) encode the proteins responsible for biosynthesis of the FeGP cofactor.^[3]^ HcgC catalyzes the methylation of 6‐carboxymethyl‐5‐methyl‐4‐hydroxy‐2‐pyridinol (**1**) to form the 3‐methylated precursor (**2**).^[4]^ HcgB catalyzes guanylylation at the 4‐position of **2** to form **3**.^[5]^ The 6‐carboxymethyl group of **3** is adenylylated by HcgE and then forms a thioester bond with a cysteine residue from HcgF to form **4**.^[6]^ Precursor **4** is further converted to the acyl ligand of the FeGP cofactor by unknown reactions.^[7]^ HcgD contains a dinuclear iron site and one of the iron ions dissociated in the presence of chelating reagents, which suggests that HcgD is an iron‐trafficking protein.^[8]^ The enzyme that functions in biosynthesis of pyridinol precursor **1** is unknown.^[3]^ Comparative genomic analysis suggested that the only proteins involved in biosynthesis of the FeGP cofactor are HcgA‐G proteins,^[3]^ thus, we speculated that HcgA or HcgG might catalyze biosynthesis of **1** or completion of the iron complex, including formation of the CO and acyl ligands.

HcgA is a member of the radical *S*‐adenosyl methionine (SAM) enzyme family and is similar to [FeFe]‐hydrogenase maturation enzymes (HydE and HydG), biotin synthase and methylornithin synthase.^[9]^ In contrast to the other radical SAM enzymes, HcgA contains a unique CX_5_CX_2_C motif rather than the standard motif (CX_3_CX_2_C) for [4Fe‐4S]‐cluster binding.^[10]^ Although a previous study indicated that HcgA contains a [4Fe‐4S] cluster and catalyzes the formation of the 5′‐deoxyadenosine from SAM in the absence of the substrate, the substrate and catalytic reaction of HcgA have not been reported.^[10a]^ The catalytic function of HcgG is unknown.^[9]^

Recently, we developed an in vitro biosynthesis assay for the FeGP cofactor using the cell extract from the Δ*hcgB*Δ*hcgC* mutated strain of *Methanococcus maripaludis*, which cannot biosynthesize precursor **3**.^[7]^ The standard in vitro biosynthesis assay contained the cell extract of the *M. maripaludis* Δ*hcgB*Δ*hcgC* mutant and the standard reaction mixture. Incubation of **3** in the in vitro biosynthesis assay under H_2_ and CO produced the FeGP cofactor, where the active [Fe]‐hydrogenase holoenzyme is formed by binding of the FeGP cofactor to the apoenzyme in the assay. Using this method, we demonstrated that **1**, **2** and **3** are the precursors of the FeGP cofactor biosynthesis.^[7]^ The in vitro biosynthesis assays indicated that the 6‐carboxymethyl group of **3** is converted to the acyl ligand and that the CO ligands are formed from external CO gas or from an unknown CO‐donating compound in the cell extract. Furthermore, these experiments revealed that an electron donor is needed for biosynthesis.

Here, we report the function of the radical SAM enzymes, HcgA and HcgG, in biosynthesis of the FeGP cofactor using in vitro complementation assays. Our data indicated that HcgA catalyzes biosynthesis of **1** and that HcgG contributes to the formation of the CO and acyl ligands to complete biosynthesis of the FeGP cofactor.

In this study, we used the cell extract from the Δ*hcgA* and Δ*hcgG* strains of *M. maripaludis* (Table S1). We confirmed the absence of the respective proteins in the cell extracts of the Δ*hcgA* and Δ*hcgG* strains by proteome analysis (Table S2). Accordingly, the cell extracts of the mutated strains did not show [Fe]‐hydrogenase activity. The in vitro biosynthesis assay solution contained the standard reaction cocktail and the following tested materials: the cell extract of the *M. maripaludis* Δ*hcgA* or Δ*hcgG* strain, the purified Hcg enzymes and/or the biosynthesis precursors (**1**, **2** or **3**). The standard reaction cocktail contained ATP/MgCl_2_, dithiothreitol (DTT), sodium dithionite, [Fe]‐hydrogenase apoenzyme from *Methanocaldococcus jannaschii*, Fe^2+^ and SAM. The gas phase of the standard assay was 50 % H_2_/50 % CO. To evaluate the in vitro biosynthesis of the FeGP cofactor, we determined the enzyme activity of the [Fe]‐hydrogenase that was constructed in the assay. For details, see the Experimental Procedures section in the Supporting Information.

We heterologously produced HcgA of *M. maripaludis* in *Escherichia coli* as a His‐tagged protein using a helper plasmid (pRKISC) to improve iron‐sulfur cluster biosynthesis (Figures S1 and S2).^[11]^ The UV/Vis spectrum of the purified HcgA was in accordance with a previous report,^[10a]^ and reduction by dithionite was observed as a decrease in absorbance at 410 nm (Figure 2a). As the heterologous production of HcgG in *Escherichia coli* resulted in the formation of inclusion bodies,^[12]^ we expressed His‐tagged HcgG *in trans* in *M. maripaludis* using an expression vector with the *Methanococcus voltae* histone promoter (Supporting Information, Experimental Procedures). Gel permeation chromatography indicated that the molecular mass of the purified HcgG was 98 kDa (homodimer of 57 kDa subunits; Figure S3). The UV/Vis spectrum of HcgG showed the presence of absorbance peaks at 350, 420, and 600 nm (Figure 2b). The addition of sodium dithionite slightly decreased the absorbance at 420 nm.

**Figure 2:**
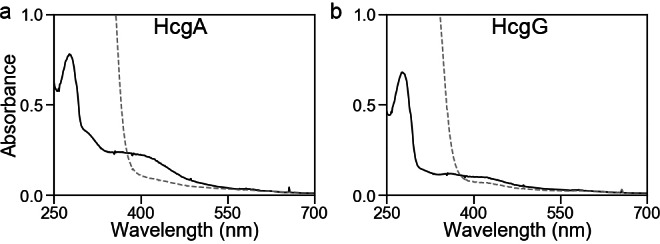
UV/Vis spectra of a) 5 mg ml^−1^ HcgA and b) 2 mg ml^−1^ HcgG as purified (solid lines) and incubated with 5 mM sodium dithionite (dashed line) in a 3 mm light‐path quartz cuvette.

Without added precursors, the in vitro biosynthesis assay with the cell extract from the Δ*hcgA* strain did not produce [Fe]‐hydrogenase activity (Figure 3a). When 10 μM of each precursor was added, we detected the formation of [Fe]‐hydrogenase activity. Complementation with **1** or **2** required GTP as the substrate for the HcgB reaction. These complementation experiments indicated that the Δ*hcgA* strain does not produce pyridinol precursor **1**, which suggested that HcgA catalyzes biosynthesis of **1**. The addition of purified HcgA to the in vitro biosynthesis assay without external precursors yielded [Fe]‐hydrogenase activity, which indicated that heterologously produced HcgA is active and that the substrate of the HcgA reaction is present in the cell extract of the Δ*hcgA* strain.

**Figure 3:**
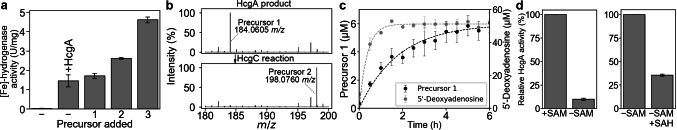
Function of HcgA. a) In vitro biosynthesis of the FeGP cofactor using the *ΔhcgA* mutant cell extract with the pyridinol precursors (**1**, **2** or **3** shown in abscissa) with HcgA (+HcgA) and without HcgA (no label). (−) No precursors added. b) MS analysis of the HcgA reaction product (precursor **1**) using the 3 kDa filtrate of the cell extract from the *M. maripaludis* Δ*hcgA* strain as the substrate (top). The HcgC reaction formed precursor **2** (bottom; see Figure 1). c) HPLC‐MS quantification of the time‐dependent reaction of HcgA, in which the production of **1** is shown as black dots and the production of 5′‐deoxyadenosine is shown as gray dots. Dashed lines indicate an exponential function fit. d) (left) Effect of removal of SAM from the **1**‐forming HcgA assay (−SAM) in comparison with the full assay containing SAM (+SAM), and (right) effect of addition of 1 mM *S*‐adenosyl homocysteine (−SAM +SAH) in the assay without SAM (−SAM). The effect is indicated as a relative activity (%). Error bars represent the standard deviation of two (panel a) or three (the other panels) measurements.

A 184.0605 *m/z* compound was produced by incubating the cell extract filtrate (3 kDa cut‐off) of the Δ*hcgA* strain with HcgA (Figure 3b and Figure S4). This mass corresponds to that of **1** (calculated 184.0604 *m/z*). The MS/MS signals of this compound are also identical to those found in chemically synthesized **1** (Figure S5). When HcgC was added to the assay, the 184 *m/z* compound disappeared and a 198.0760 *m/z* signal was produced, which is identical to the signal of **2** (calculated 198.0682 *m/z*; Figure 3b). This result indicated that the 184 *m/z* compound is the substrate of HcgC and further confirmed that HcgA catalyzes the formation of **1** from a filtrated compound in the cell extract. The amount of **1** in the assay increased in a time‐dependent manner by the HcgA reaction (Figure 3c). Along with the formation of **1**, the byproduct of the radical SAM enzyme reaction, 5′‐deoxyadenosine (measured 252.1085 *m/z*, calculated 252.1091 *m/z*) was produced (Figure 3c). We also detected the formation of 5′‐deoxyadenosine by the HcgA‐catalyzed reaction in the absence of the cell extract filtrate (Figure S6). Unexpectedly, the kinetic analysis indicated that 5′‐deoxyadenosine is produced by the HcgA reaction much faster than precursor **1** and increase of 5′‐deoxyadenosine in the assay was slowed down before stopping of the production of **1** (Figure 3c). One possible explanation of this discrepancy of stoichiometry is the existence of an additional enzyme in the cell extract filtrate. However, we did not detect any *M. maripaludis* proteins by mass spectrometric proteome analysis of the cell extract filtrate. The HcgA activity decreased in the absence of external SAM in the assay (Figure 3d). The HcgA activity was inhibited by a radical SAM enzyme inhibitor, *S*‐adenosyl homocysteine (SAH; Figure 3d and Figure S6).

Based on a previous labeling study, it was predicted that β‐alanine or aspartate reacts with 2,3‐dihydroxy‐4‐oxo‐pentanoate to form **1**.^[13]^ We tested the HcgA reaction in the presence of [^15^N,^13^C]‐β‐alanine or a mixture of twenty essential [^15^N]‐amino acids using the Δ*hcgA* cell extract, SAM and HcgA. These reactions did not produce ^15^N‐ and/or ^13^C‐labeled **1**, which does not support the previous proposal (Figures S7 and S8).

To test the catalytic function of HcgG, we performed the in vitro biosynthesis assay using the cell extract from the Δ*hcgG* strain of *M. maripaludis*. The addition of **3** into the in vitro biosynthesis assay did not complement the biosynthesis activity (Figure 4a), while the addition of purified HcgG recovered the in vitro biosynthesis activity even in the absence of external **3**. Simultaneous addition of HcgG and **3** resulted in higher activity. These results indicated that HcgG catalyzes a reaction after the formation of **3**. Under 100 % H_2_, the activity obtained was similar to that obtained under 50 % H_2_/50 % CO (Figure 4a). This was unexpected because previous studies showed that in vitro biosynthesis in the Δ*hcgB*Δ*hcgC* mutant under the 100 % H_2_ gas phase decreased the activity to approximately 30 % of that obtained under 50 % H_2_/50 % CO.^[7]^ This result suggests that the addition of purified HcgG to the in vitro biosynthesis assay accelerates the biosynthesis reaction using a CO‐donor compound in the cell extract. To confirm this prediction, we titrated HcgG into the assay mixture. Indeed, the biosynthesis activity of HcgG under 100 % H_2_ needed more HcgG in the assay to reach the same activity obtained in the presence of CO (Figure 4b). These findings indicate that HcgG contributes to the following catalytic activities: an external CO‐dependent activity with a higher reaction rate and a slower CO‐independent activity that utilizes a compound in the cell extract as a source of CO.

**Figure 4:**
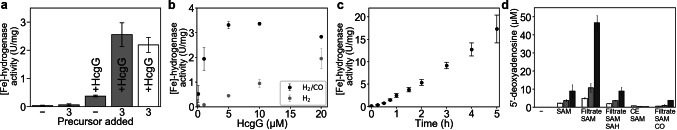
Function of HcgG. a) In vitro biosynthesis of the FeGP cofactor using the cell extract of the *ΔhcgG* strain. The assays were performed in the absence (−) or presence of precursor **3** (3) with HcgG (+HcgG) and without HcgG (no label) under a 50 % H_2_/50 % CO atmosphere (gray bars) or 100 % H_2_ atmosphere (open bar). b) In vitro biosynthesis of the FeGP cofactor in the cell extract from the *ΔhcgG* strain with different amounts of HcgG under a 50 % H_2_/50 % CO atmosphere (black dots) or 100 % H_2_ atmosphere (light gray dots). c) Time‐dependent increase in the [Fe]‐hydrogenase activity in in vitro biosynthesis of the FeGP cofactor using the cell extract from the *ΔhcgG* strain from precursor **2** in the presence 20 μM HcgG and 5 mM GTP. d) HPLC‐MS quantification of 5′‐deoxyadenosine after incubation with HcgG for 0 h (open bar), 1 h (light gray bar) and 6 h (black bar) in the presence of SAM, the cell extract (CE) of the *ΔhcgG* strain, its 3 kDa cell extract filtrate (Filtrate) and/or SAH. A negative control with only 50 mM Tris/HCl pH 7.4 was also tested (−). The experiments were performed under a 95 % N_2_/5 % H_2_ atmosphere except for one condition with the filtrate and SAM under a 47.5 % N_2_/2.5 % H_2_/50 % CO atmosphere (CO). Error bars correspond to the standard deviations of three independent measurements.

The kinetics of the HcgG‐dependent reaction were determined in the cell extracts from the Δ*hcgG* strain (Figure 4c). In the kinetic samples using the cell extract, we did not detect 5′‐deoxyadenosine, probably due to 5′‐deoxyadenosine decomposing activity.^[14]^ We detected the formation of 5′‐deoxyadenosine from SAM by the HcgG reaction in the absence of the cell extract (Figure 4d). The addition of the cell extract filtrate (3 kDa cut‐off) stimulated the production of 5′‐deoxyadenosine. These results indicated that HcgG performs a radical SAM reaction. Accordingly, SAH inhibited the 5′‐deoxyadenosine‐forming reaction (Figure 4d). This radical SAM reaction is probably involved in CO‐ligand formation from a cellular material, which is reminiscent of the production of CO‐ligands by HydG in [FeFe]‐hydrogenase maturation.^[15]^ CO in the gas phase inhibited the formation of 5′‐deoxyadenosine, which indicated that the radical SAM reaction catalyzed by HcgG is inhibited by CO. A structural model of HcgG from *Methanocaldococcus jannaschii* is available from the AlphaFold database.^[16]^ The N‐terminal domain of the model structure shows similarities to HydG, which catalyzes the CO‐forming reaction (Figure S9). Notably, the C‐terminal domain of the modeled HcgG shows similarities to the N‐terminal domain of a homolog of [Fe]‐hydrogenase (HmdII), where the FeGP cofactor binds.^[17]^

In this study, we investigated the enzymatic activity of HcgA and HcgG in biosynthesis of the FeGP cofactor. HcgA catalyzes a radical SAM reaction to form **1** from an unknown precursor present in the cell extract. HcgG was obtained by homologous production in *M. maripaludis*. In vitro biosynthesis using the Δ*hcgG* strain indicated that HcgG contributes to CO ligand formation from a CO‐donor substrate and/or CO gas, and the completion of biosynthesis of the FeGP cofactor, including the acyl ligand formation. However, it cannot be excluded that other common enzymes in the cell extract work together with HcgG as recently observed in the role of the glycine cleavage system in [FeFe]‐hydrogenase maturation.^[18]^ To solve this problem, we need to establish a cell‐extract‐free in vitro biosynthesis. Although the substrates of HcgA and HcgG are still unknown, the in vitro biosynthesis assay allows us to purify and identify the unknown compounds. We also established a method to prepare spectroscopically and structurally sufficient amounts of the active forms of HcgA and HcgG, which paves the way for further characterizing these novel radical SAM enzymes.

## Conflict of interest

The authors declare no conflict of interest.

## Supporting information

As a service to our authors and readers, this journal provides supporting information supplied by the authors. Such materials are peer reviewed and may be re‐organized for online delivery, but are not copy‐edited or typeset. Technical support issues arising from supporting information (other than missing files) should be addressed to the authors.

Supporting Information

## Data Availability

The data that support the findings of this study are available from the corresponding author upon reasonable request.
