# Supplementary material for: The Function of Two Radical‐SAM Enzymes, HcgA and HcgG, in the Biosynthesis of the [Fe]‐Hydrogenase Cofactor
Source: Angew Chem Int Ed Engl. 2022 Nov 17;61(50):e202213239. doi: 10.1002/anie.202213239 (PMC10100467; doi:10.1002/anie.202213239)
Supplement: Supplementary file 1 — Supporting Information [file ANIE-61-0-s001.pdf]

Angewandte Chemie
Eine Zeitschrift der Gesellschaft Deutscher Chemiker

Supporting Information

The Function of Two Radical-SAM Enzymes, HcgA and HcgG, in the Biosynthesis of the [Fe]-Hydrogenase Cofactor

*F. J. Arriaza-Gallardo, S. Schaupp, Y.-C. Zheng, M. F. Abdul-Halim, H.-J. Pan, J. Kahnt, G. Angelidou, N. Paczia, X. Hu, K. Costa, S. Shima**

Table of Contents

Experimental Procedures 3
Heterologous production and purification of HcgA 3
Construction of a *M. maripaludis* strain for the expression of His_6 tagged HcgG. 3
Homologous production and purification of HcgG 3
Preparation of the cell extract from *Methanococcus maripaludis* for in vitro biosynthesis 3
In vitro biosynthesis of the FeGP cofactor 4
In vitro reactions of HcgA or HcgG 4
Proteome analysis 4
Determination of compounds **1**, **2** and 5'-deoxyadenosine. 4
Determination of the FeGP cofactor by ESI-MS 4
Figure S1. Sequences of the genes encoding HcgA and HcgG used in this work 6
Figure S2. Gel permeation chromatography of HcgA after Ni affinity purification. 7
Figure S3. Gel permeation chromatography of HcgG after Ni affinity purification. 7
Figure S4. HPLC-ESI-MS extracted ion chromatograms (EIC) at 184.0604 *m/z*.. 8
Figure S5. HPLC-ESI-MS/MS analysis of the compound with 184.0604 *m/z*. 8
Figure S6. HPLC-MS quantification of 5'-deoxyadenosine after incubation of HcgA. 9
Figure S7. Precursor **1** produced by the HcgA reaction with ^{15}N-labelled amino acids mixture 10
Figure S8. Precursor **1** produced by the HcgA reaction with [^{15}N,^{13}C]-ß-alanine 10
Figure S9. Structural prediction of HcgG by AlphaFold. 11
Figure S10. Multiple sequence alignments of HcgG from different microorganisms. 12
Table S1. Strains used in this work. 13
Table S2. Proteome analysis of the ∆hcg mutants. 13
References. 14
Author contributions. 14

Experimental Procedures

Heterologous production and purification of HcgA

Escherichia coli C41(DE3) cells were transformed with a plasmid (pRKISC) for overexpression of Fe-S cluster proteins,[1] and then further transformed with the expression plasmid pET28b(+) containing the synthesized *M. maripaludis hcgA* gene with a N-terminal His_6 tag (GenScript), whose codon usage was optimized for expression in *E. coli* (Figure S1) and cloned between the XhoI and NdeI restriction sites. The transformant was grown at 37 °C in Terrific Broth (1.2% Tryptone, 2.4% yeast extract, 0.5% glycerol and 89 mM potassium phosphate) supplemented with 50 µg/ml kanamycin and 10 µg/ml tetracycline. At optical density 600 (OD_{600}) = 1.0, the expression of the *hcgA* gene was induced with 1 mM isopropyl ß-*D*-thiogalatopyranoside and supplemented with 0.12 g/L cysteine dihydrochloride, 0.1 g/L iron (II) sulfate, 0.1 g/L iron citrate and 0.1 g/L ferric ammonium citrate (final concentrations) and cultivated for 18 hours at 20 °C. After the incubation, the cells were harvested by centrifugation using Avanti JXN-26 centrifuge with JLA-10.500 rotor at 7,300 rpm for 30 min at 4 °C and stored at –20 °C. All purification steps were performed anaerobically in an anaerobic chamber (Coy Laboratories, Grass Lake, MI, USA). The frozen cells containing HcgA were resuspended in 20 mM sodium phosphate buffer pH 7.4 containing 0.5 M sodium chloride, 5% glycerol and 20 mM imidazole (Buffer A) and disrupted on ice by sonication for 10 min using SONOPULS GM200 (Bandelin) with KE76 tip with 50 cycles. The supernatant was collected by centrifugation in a Sorvall WX Ultra centrifuge with a T-647.5 rotor at 30,000 rpm for 30 min at 4 °C and loaded onto a Ni^{2+}-charged HiTrap chelating column (cytiva, Freiburg im Breisgau, Germany) equilibrated with buffer A. The column was washed with buffer A and eluted with a linear gradient of imidazole from 20 to 500 mM in 20 mM sodium phosphate buffer pH 7.4 containing 0.5 M NaCl and 5% glycerol (w/v). The protein fractions were concentrated with a centrifugal filter unit (Merck Millipore, Darmstadt, Germany) and loaded onto a HiPrep 26/10 desalting column (GE Healthcare, Uppsala, Sweden) equilibrated with 50 mM 3-(N-morpholino)propanesulfonic acid (MOPS)/NaOH buffer pH 7.4 containing 0.5 M NaCl and 20% glycerol (w/v). The protein was further purified by gel permeation chromatography using a HiPrep Sephacryl S-300 HR (GE Healthcare, Uppsala, Sweden) equilibrated with 50 mM MOPS/NaOH buffer pH 7.4 containing 0.5 M NaCl and 20% glycerol (w/v). The fractions were pooled and concentrated using a 30 kDa centrifugal filter unit. The protein was frozen in liquid N_2 and stored at −20 °C.

Construction of a *M. maripaludis* strain for the expression of His_6 tagged HcgG.

M. maripaludis hcgG gene was amplified with the primer pairs 0125_Fg (CCATCACATCGAAGGTCGTGGGCCCATGAAAGAACTCATAAAAAATTCATTAAATG) and 0125_Rg (TTTTATGACCTACAGATCTCCTAGGTTAAAGTAATGATACGGCATC), and cloned into pLW40neo digested with ApaI and AvrII as previously described.[2] The resulting plasmid (pWL40neo*HishcgG*) was transformed into *M. maripaludis* ∆*upt* (Mm901) by the polyethylene glycol (PEG) method as previously described with PEG 8000 (Millipore Sigma).[3]

Homologous production and purification of HcgG

M. maripaludis transformed with pWL40neo*HishcgG* was grown in 10 L glass fermenters with 9 L McCas medium[4] supplemented with 1 g/L neomycin, under constant gas flow of $H_2/CO_2/H_2S$ (80%/20%/0.1%) at 1.5 L/min. Cells were grown to an OD at 660 nm = 2. The fermenters were then cooled with ice water and the cells were harvested anaerobically by a continuous-flow centrifuge equipped with a Heraeus 3049 continuous flow rotor at 15,000 rpm at 4 °C under N_2 atmosphere. The rotor was then transferred to an anaerobic chamber and the cells were resuspended in the residual medium in the rotor, then the cells were further centrifuged using an Avanti JXN-26 centrifuge with JLA-10.500 rotor at 15,000 rpm for 30 min at 4 °C. The cells were weighted and resuspended in 2-fold volume per weight of lysis buffer (50 mM Tris pH 7.4, 5 mM $MgCl_2$, 0.5‑2.5 U/ml DNAse I), aliquoted, flash frozen in liquid N_2 and stored anaerobically at −75 °C. All purification steps were done anaerobically. Cell resuspension was thawed and disrupted using a Thermo IEC FRENCH® Press in a 40K cell at 20,000 psi for five cycles. Clear cell-free extracts were obtained by centrifugation in a Sorvall WX Ultra centrifuge with a T-647.5 rotor at 30,000 rpm for 30 min at 4 °C and subsequent filtration by 0.45 µm filters. The resulting supernatant was loaded onto two 5-ml Ni^{2+}-charged HiTrap chelating column (cytiva, Freiburg im Breisgau, Germany) connected in series and equilibrated with 20 mM sodium phosphate buffer pH 7.4 containing 0.5 M sodium chloride, 5% glycerol (w/v) and 20 mM imidazole (Buffer A). The column was washed with buffer A and eluted with a linear gradient of imidazole from 20 to 500 mM in 20 mM sodium phosphate buffer pH 7.4 containing 0.5 M NaCl and 5% glycerol (w/v). The protein fractions were concentrated with a centrifugal filter unit (Merck Millipore, Darmstadt, Germany) and loaded onto a HiPrep Sephacryl S-300 HR (GE Healthcare, Uppsala, Sweden) equilibrated with 50 mM MOPS/NaOH buffer pH 7.4 containing 0.5 M NaCl, 20% glycerol (w/v), and 2 mM dithiothreitol. After SDS-PAGE, the fractions containing HcgG were pooled, concentrated using a 30 kDa centrifugal filter unit. The sample was frozen in liquid N_2 and stored at −75 °C.

Preparation of the cell extract from *Methanococcus maripaludis* for *in vitro* biosynthesis

Methanococcus maripaludis ∆*hcgA* and ∆*hcgG* strains were cultivated in the 37 °C cultivation room using a medium with sodium formate as substrate under 80% N_2 / 20% CO_2, in which 100 mM Tris was added as buffer component.[5] Cultivation was performed in 5 L or 500 mL scale until an OD at 660 nm of 0.6–0.8 as described previously.[6] The actively growing cells were anaerobically harvested by a continuous-flow centrifuge (Heraeus 3049 continuous flow rotor at 15,000 rpm at 4°C), resuspended in medium again and sedimented by centrifugation (Beckmann JLA 10.500 rotor at 7,300 rpm and 4°C). The use of the culture medium for resuspension aimed to avoid lysis of the cells in low salt concentration buffer solutions. The cell pellets were finally anaerobically resuspended in a low salt concentration lysis buffer: 50 mM Tris/HCl pH 7.5, 5 mM $MgCl_2$ and 2.5 U/mL DNaseI, to a final concentration of 0.5 g cells/mL buffer. One mL aliquots were frozen in liquid N_2 and stored until use at −20°C. The frozen samples

were anaerobically thawed on ice. Unbroken cells and membrane particles were removed by ultracentrifugation using a Sorvall TFT-80.4 rotor at 37,000 rpm and 4°C for 0.5 h. This supernatant is designated as cell extract and used for *in vitro* biosynthesis assay.

In vitro **biosynthesis of the FeGP cofactor**

In vitro biosynthesis of the FeGP cofactor was performed as previously described.[6] Briefly, 200 µL cell extract of *M. maripaludis* was supplemented with 1 mM $Fe(SO_4)_2(NH_4)_2$, 1 mM DTT, 2 mM sodium dithionite, 5 mM $MgCl_2$, 2 mM SAM, 5 mM ATP, 10 µM precursors and 10 µM [Fe]-hydrogenase apoenzyme (final concentrations) under 50% H_2 /50%CO, if no other conditions are mentioned. In the standard assays of *in vitro* biosynthesis using the cell extract from the Δ*hcgA* or Δ*hcgG* strains, 20 µM HcgA or 20 µM HcgG were added to the assay, respectively. In *in vitro* biosynthesis from precursors **1** and **2**, 5 mM GTP were added to convert the precursors to **3** by endogenous HcgB. The solution was transferred to a vial containing 50% H_2 / 50% CO or otherwise described atmosphere and sealed with a rubber stopper. The reaction mixtures were incubated at 40 °C for 1 hour. In the case of kinetic analysis of the HcgG reaction was performed at 20 °C (Figure 4c). The [Fe]-hydrogenase activity was determined in 1 cm light-path quartz cuvette with 0.7 ml sample containing 20 µM methylene-H_4MPT as the substrate in 120 mM potassium phosphate pH 6.0 amended with 1 mM EDTA at 40 °C under N_2 as previously described.[7] The formation of methenyl-H_4MPT^+ was determined by measuring the increase of the absorbance at 336 nm. The extinction coefficient of methenyl-H_4MPT^+ is 21.6 $mM^{-1}cm^{-1}$. The reaction was started by the addition of the sample tested after dilution with the assay buffer. One unit of the activity is the amount of enzyme producing 1 µmol of methenyl-H_4MPT^+ per min.

In vitro **reactions of HcgA or HcgG**

Cell extract of *M. maripaludis* was filtrated with a 3 kDa cut-off filter to remove proteins. To determine the production of 5'-deoxyadenosine by the HcgA and HcgG reactions or the production of precursor **1** in the HcgA reaction, 200 µL reaction mixtures were prepared, which included 170 µL cell extract, 3 kDa filtrated cell extract or lysis buffer (50 mM Tris/HCl pH 7.4, 5 mM $MgCl_2$, 0.5-2.5 U/ml DNAse I), in addition 20 µM HcgA or HcgG, 5 mM sodium dithionite, 2.5 mM SAM and 1 mM of the other components described specifically in the figure legends. The resulting reaction mixtures were sealed with 100 µL mineral oil to avoid evaporation, then incubated at 37 °C overnight or for the time period described, either under an 95% N_2/5% H_2 atmosphere or 47.5% N_2/2.5% H_2/50% CO atmosphere. At the time indicated in the figures, 50 µL aqueous samples were taken, and quenched by mixing with 200 µL methanol, the resulting mixtures were incubated at 40 °C for 15 minutes for extraction of small components, and then subjected to 0.45 µm filtration (Ultrafree-MC Centrifugal Filter, Millipore, Germany), evaporated to dryness by using a Heraeus Centrivac aerobically. The resulting residuals were dissolved in 50 µL distilled water and stored at −20 °C until further LC-MS analysis.

For the^{15}N-labeling experiment: Cell Free Amino Acid Mixture-^{15}N solution* (Sigma-Aldrich) containing 60 mM aspartate, 35 mM threonine, 35mM serine, 40 mM glutamate, 20 mM proline, 100 mM glycine, 100 mM alanine, 40 mM valine, 10 mM methionine, 30 mM isoleucine, 45 mM leucine, 10 mM tyrosine, 16 mM phenylalanine, 5 mM histidine, 15 mM lysine, 10 mM arginine, 20 mM glutamine, 20 mM asparagine, 20 mM tryptophane and 20 mM cysteine, or 100 mM [$^{13}C_3$-$^{15}N_1$]-ß-alanine (Sigma-Aldrich) was used. The [^{15}N]-amino-acid mixture or [^{13}C-^{15}N]-ß-alanine was mixed with the same volume of the 3-kDa filtrate of the cell extract from *M. maripaludis* Δ*hcgA* strain and used for the reaction with the standard *in vitro* reaction of HcgA. To check the effect of the other cellular enzymes, the labelled amino acids were mixed with the same volume of the cell extract and incubated for 2 hours in the anaerobic tent at room temperature. After centrifugation, the supernatant was filtrated with 3-kDa cut-off filter and used for the *in vitro* HcgA reaction.

Proteome analysis

Cell pellets were lysed and reduced by 5 mM tris(2-carboxyethyl)phosphine (TCEP) in the presence of 2% deoxycholate (DOC) at 90 °C for 10 minutes. After that, it was incubated at 25 °C for 30 minutes in 100 mM ammonium bicarbonate pH 8.2 and 10 mM iodacetamide (IAA) and then digested overnight at 30 °C with trypsin, MS approved (Serva). Before LC-MS analysis, samples were desalted using C18 microspin columns (Nest Group) according to the manufacturer's instructions. Dried and reconstituted peptides were then analyzed using liquid-chromatography-mass spectrometry carried out on a Orbitrap Exploris 480 instrument connected to an Ultimate 3000 RSLC nano and a nanospray ion source (Thermo Scientific). Peptide separation was performed on a reverse phase HPLC column (75 µm x 42 cm) packed with C18 resin (2.4 µm; Dr. Maisch) with a 90-minute gradient (formic acid / acetonitrile). MS data were searched against an in-house *Methanococcus maripaludis* S2 protein database using SEQUEST embedded into Proteome Discoverer 1.4 software (Thermo Scientific).

Determination of compounds 1, 2 and 5'-deoxyadenosine

Determination of compounds was performed using two different types of HResLC-MS. In both cases, the chromatographic separation was performed using a Kinetex EVO C18 column (150 × 1.7 mm, 3 µm particle size, 100 Å pore size, Phenomenex) connected to a guard column of similar specificity (20 × 2.1 mm, 5 µm particle size, Phenomoenex) a constant flow rate of 0.2 mL/min with mobile phase A being 0.1% formic acid in water and phase B being 0.1% formic acid methanol (Honeywell, Morristown, New Jersey, USA) at 40 °C. The injection volume was 5 µL. The mobile phase profile consisted of the following steps and linear gradients: 0 – 0.5 min constant at 5% B; 0.5 – 4 min from 5 to 90% B; 4 – 5 min constant at 90% B; 5 – 5.1 min from 90 to 5% B; 5.1 – 10 min constant at 5% B. For determinations, a Thermo Scientific I-DX Orbitrap mass spectrometer was used. Ionization was performed using a high temperature electro spray ion source at a static spray voltage of 3300 V, Sheath gas at 50 (Arb), Auxiliary Gas at 25 (Arb), and Ion

transfer tube and Vaporizer at 325 and 300 °C. Data dependent MS/MS measurements were conducted applying an orbitrap mass resolution of 120 000 using quadrupole isolation in a mass range of 100 – 600 and combining it with a high energy collision dissociation (HCD). HCD was performed on the five most abundant ions per scan with a relative collision energy of 15%. Fragments were detected using the orbitrap mass analyzer at a predefined mass resolution of 30 000. Dynamic exclusion with and exclusion duration of 2.5 seconds after 1 scan with a mass tolerance of 10 ppm was used to increase coverage.

HcgA from *M. maripaludis*

Protein sequence
MGSSHHHHHHSSGLVPRGSH**M**IFSKIRGNFQDLKDGKIDVKQGLITKSDAIELFNIKNWKDYLELFSIASEVRDVFKTEIEIT
STVHVTNICSVNPKCKYCGFAAGTSSEGYVKPFRSDDDQIKSSSVAIENSGIKRVSCSSGHGYNGKEVIRALKAVKSASNLEV
LVNAGADLTEECILELKKYKIDTICCNLETTNKTVFNNVKPGENLEDRINVCKMVKKHGVELSSGLLIGIGETYEDRVEHLFF
LKELDVEEIPIMGFNPYKETPMETCPKCSAIEQAKTIAIVRLLFPDIRITSPTPTMGPELSQFALLGGASNIATVIPDNHPMN
IKGVGNPKTGNLKDVICMIKELGLTPKLN

Codon optimized sequence
CATATGATCTTCTCAAAAATTCGTGGCAACTTCCAAGACCTGAAAGACGGCAAAATCGACGTGAAACAAGGCCTGATTACCAA
AAGCGACGCTATCGAACTGTTCAACATCAAAAACTGGAAAGACTATCTGGAACTGTTTTCGATTGCGAGCGAAGTTCGTGATG
TCTTCAAAACCGAAATTGAAATCACCAGCACCGTGCATGTTACGAACATCTGCTCAGTGAATCCGAAATGCAAATATTGTGGC
TTTGCGGCCGGTACCAGCTCTGAAGGCTACGTTAAACCGTTCCGCAGCGATGACGATCAGATTAAAAGTTCCTCAGTGGCCAT
TGAAAACTCTGGCATCAAACGTGTTAGTTGCAGCAGCGGCCACGGTTATAATGGTAAAGAAGTTATTCGCGCGCTGAAAGCCG
TCAAATCCGCATCAAACCTGGAAGTCCTGGTGAATGCAGGCGCTGACCTGACCGAAGAATGTATCCTGGAACTGAAAAAATAC
AAAATCGATACCATCTGCTGTAACCTGGAAACCACGAACAAAACGGTGTTCAACAACGTTAAACCGGGTGAAAACCTGGAAGA
TCGTATCAACGTCTGTAAAATGGTGAAAAAACATGGCGTGGAACTGTCTAGTGGTCTGCTGATTGGCATCGGTGAAACCTATG
AAGACCGCGTCGAACACCTGTTTTTCCTGAAAGAACTGGATGTGGAAGAAATTCCGATCATGGGTTTCAACCCGTACAAAGAA
ACCCCGATGGAAACGTGCCCGAAATGTTCTGCAATTGAACAGGCTAAAACCATTGCGATCGTTCGTCTGCTGTTTCCGGACAT
TCGCATTACGAGCCCGACCCCGACGATGGGCCCGGAACTGTCGCAATTCGCCCTGCTGGGCGGTGCCAGCAACATTGCAACCG
TTATCCCGGATAACCATCCGATGAATATCAAAGGCGTCGGTAATCCGAAAACGGGTAACCTGAAAGATGTTATTTGTATGATT
AAAGAACTGGGTCTGACGCCGAAACTGAACTAACTCGAG

HcgG from *M. maripaludis*
Protein sequence
MHHHHHHIEGRCP**M**KELIKNSLNDLDSAMELRELVINKINNQKLTESDIIEIVDTVDDLSFEDTQKLGSIFRRFPLGCDLLEI
GVGPCSSSLTLPQFIENCVFTDHMGFPIHLCGYALADIAEKEGLTPIEVMNEVYNNVEVPLDLDHFGRFGPMRFPKEISHCMG
DCYYNGPPYKGCPRDRIHKRLITKEREYSNEFGEWIKKSATVCVNVVEEQGGGDHGADISEMEDVAKAAQKFGRGVEGIFHIG
DGYEDLITGLKACNDLDVDVLVIEGGPFNRSKDRLKDFAKSVAVSRILVKGGVVATNGAYEDECRVGLRSGLNVILSGFSGNH
HGYMCGYNIKEARRNNFGLPRVLKIMKEEAEKINICIANRELLKVLARSSRFLNHNENHLVYPSMIGDYFIGDAHWVSITNSK
MYNAPYFGKTLDSLEEELNCDKVGVLGGRYISWGIADALKPEELYVSDVDPWVEHATVKILNDNGINAYACNGNDKKALESAE
KSVITTMIPEIVLRIKNKFDAVSLL

Figure S1. Sequences of the genes encoding HcgA and HcgG used in this work. Starting methionine from the non-tagged protein in bold.

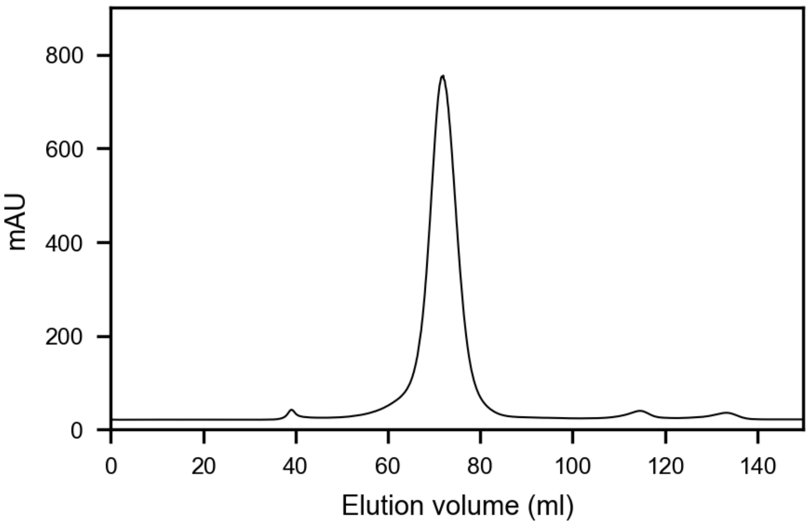

Figure S2. Gel permeation chromatography of HcgA after Ni affinity purification. The protein eluted at 71 ml, corresponding to 63 kDa by comparing the elution volume to a gel filtration standard (Bio-Rad).

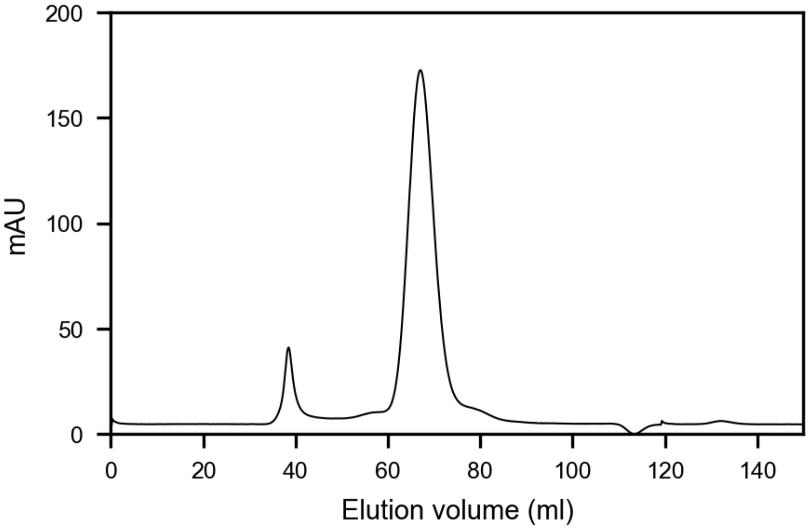

Figure S3. Gel permeation chromatography of HcgG after Ni affinity purification. The protein eluted at 66 ml, corresponding to 98 kDa by comparing the elution volume to a gel filtration standard (Bio-Rad).

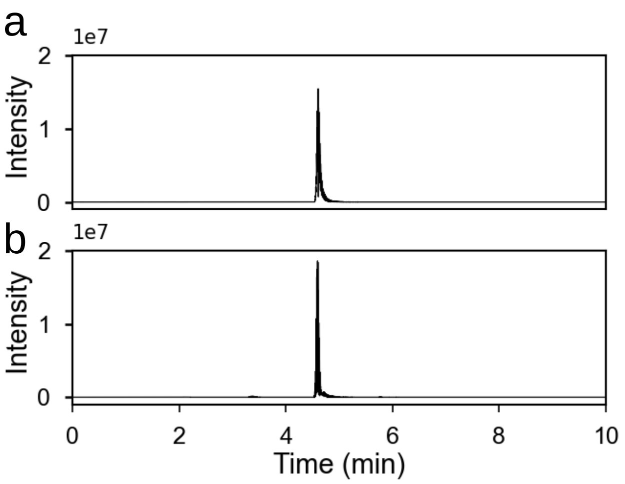

Figure S4. HPLC-ESI-MS extracted ion chromatograms (EIC) at 184.0604 *m/z*. The chemically synthesized precursor **1** (**a**) and the product of the HcgA reaction with the 3-kDa filtrate (**b**). In the reaction without HcgA, the signal was not detected.

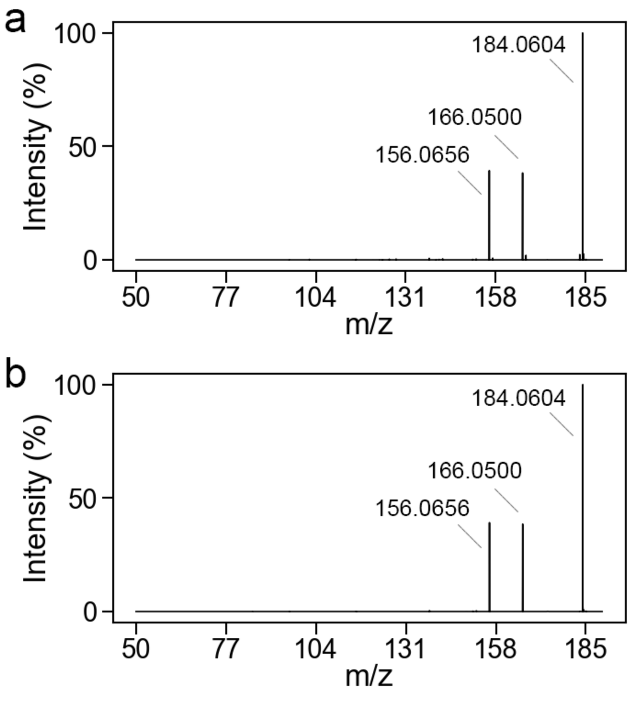

Figure S5. HPLC-ESI-MS/MS analysis of the compound with 184.0604 *m/z*. The signal produced after the reaction with HcgA (**a**) and the chemically synthesized precursor **1** (**b**).

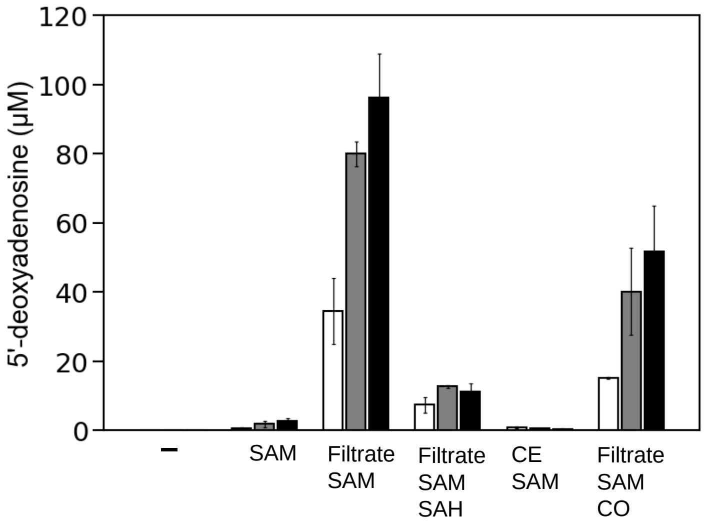

Figure S6. HPLC-MS quantification of 5'-deoxyadenosine after incubation with HcgA. Incubation was performed for 0 h (open bar), 1 h (light gray bar) and 6 h (black bar) in the presence of SAM, the cell extract (CE) of the Δ*hcgA* strain, its 3-kDa filtrate (filtrate) and/or SAH. The experiments were performed under 95% N_2 /5% H_2 atmosphere except for one condition with the filtrate and SAM under 47.5% N_2/2.5% H_2/50% CO atmosphere. Error bars correspond to the standard deviations of three independent samples.

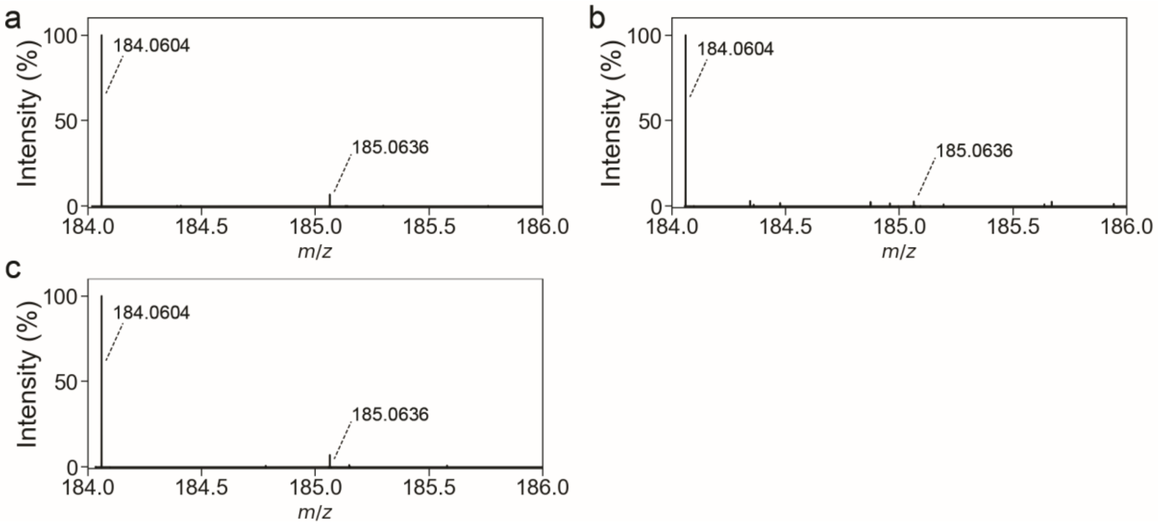

Figure S7. Precursor **1** produced by the HcgA reaction with ^{15}N-labelled amino acids mixture. MS spectra at the retention time of precursor **1** on HPLC are shown. (**a**) Reaction product incubated without labelled compounds. 184.0604 *m/z* and 185.063 *m/z* signals correspond to non-labelled precursor **1** and the natural ^{13}C-isotope of precursor **1**, respectively. (**b**) Reaction product with [$^{15}N_1$]-labelled amino acids and the cell extract filtrate. (**c**) Reaction product with [$^{15}N_1$]-labelled amino acids after incubation in the cell extract.

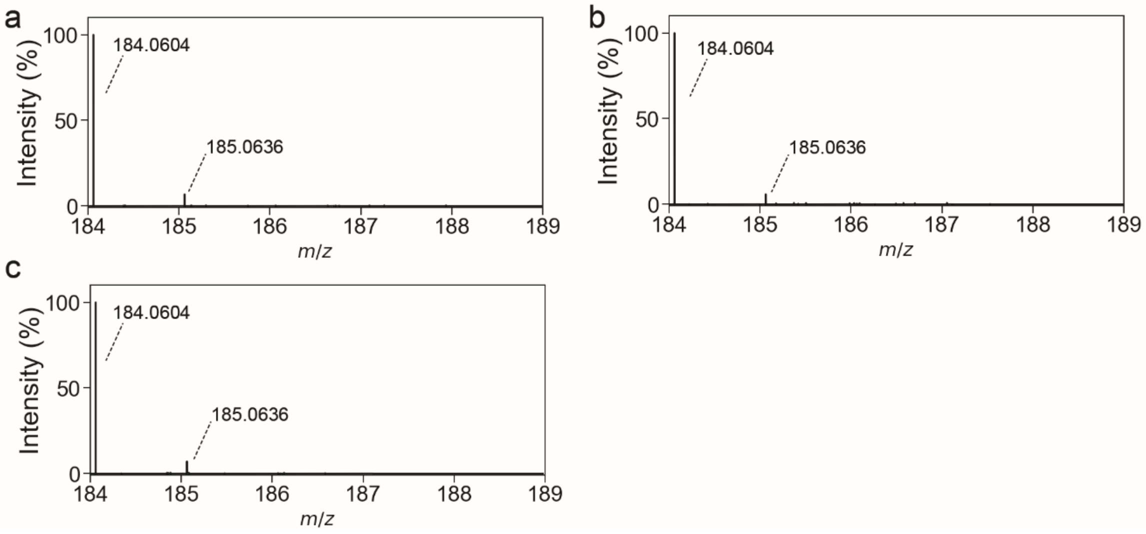

Figure S8. Precursor **1** produced by the HcgA reaction with [^{15}N,^{13}C]-ß-alanine. MS spectra at the retention time of precursor **1** on HPLC are shown. (**a**) Reaction product without labelled compounds. 184.0604 *m/z* and 185.063 *m/z* signals correspond to non-labelled precursor **1** and the natural ^{13}C-isotope of precursor **1**, respectively. (**b**) The reaction product with [$^{13}C_3$,$^{15}N_1$]-ß-alanine and the cell extract filtrate. (**c**) Reaction product with [$^{13}C_3$,$^{15}N_1$]-ß-alanine pre-incubated with the cell extract. A full incorporation of labelled [$^{13}C_3$,$^{15}N_1$]-ß-alanine should yield an 188.068 *m/z*.

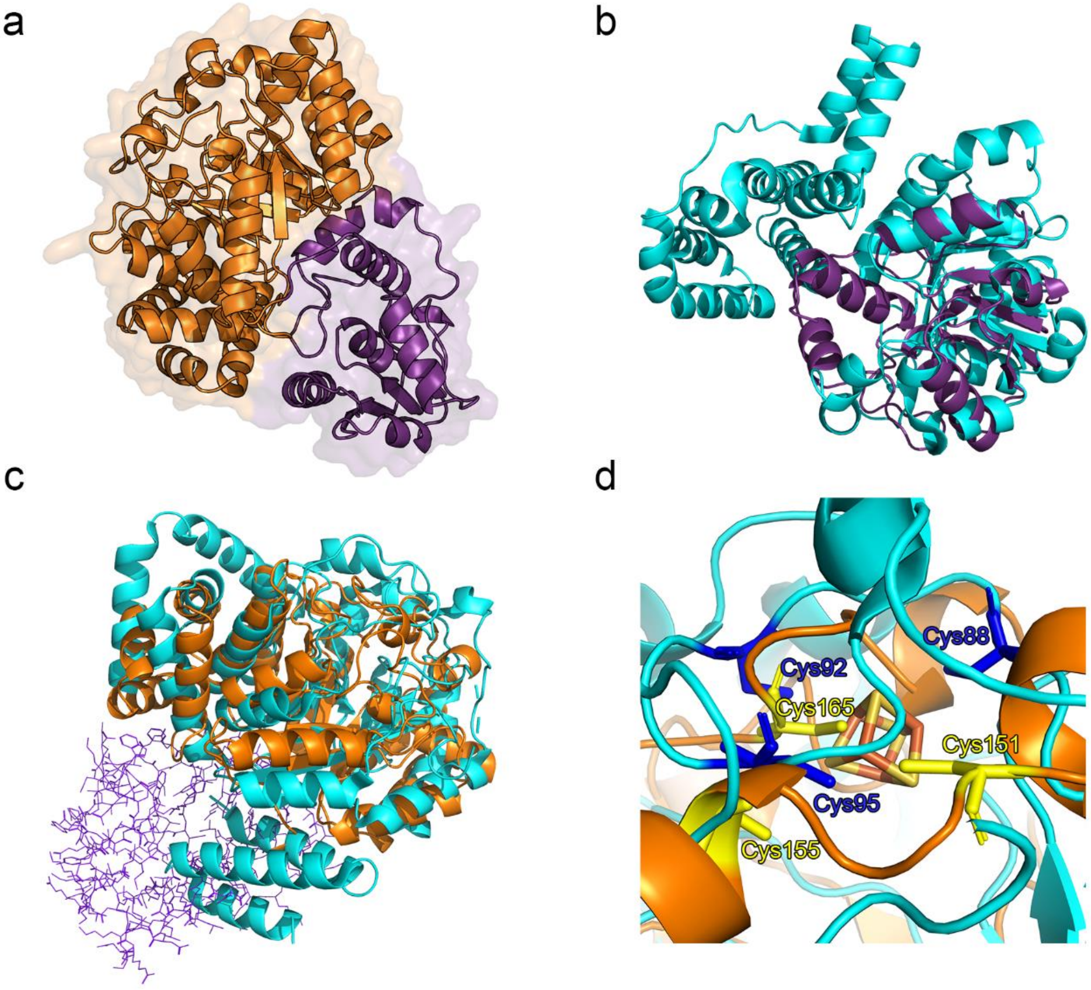

Figure S9. Structural prediction of HcgG by AlphaFold. (a) The whole structure of the AlphaFold model of HcgG from *Methanocaldococcus jannaschii*, the N-terminal (orange) and C-terminal (purple) domains are shown in cartoon model with transparent surface. (b) Structural alignment of the C-terminal domain of the HcgG model (purple) and HmdII from *M. jannaschii* (cyan) (PDB ID: 6HUX)[8] with RMSD = 2.99 Å. (c) Alignment of the N-terminal AlphaFold model of HcgG from *M. jannaschii* (orange) to the crystal structure of HydG from *Carboxydothermus hydrogenoformans* (PDB ID: 4RTB) (cyan) with RMSD = 5.77 Å. The C-terminal domain of the HcgG model is depicted in purple lines. (d) The zoom-up view of the [4Fe-4S]-cluster binding region of the HydG (cyan) with the three binding cysteine residues in blue, which is aligned with the AlphaFold model of HcgG (orange) with the conserved cysteine residues at the possible [4Fe-4S]-binding site in yellow. For sequence alignments, see Figure S10. The structure model is made by PyMOL.

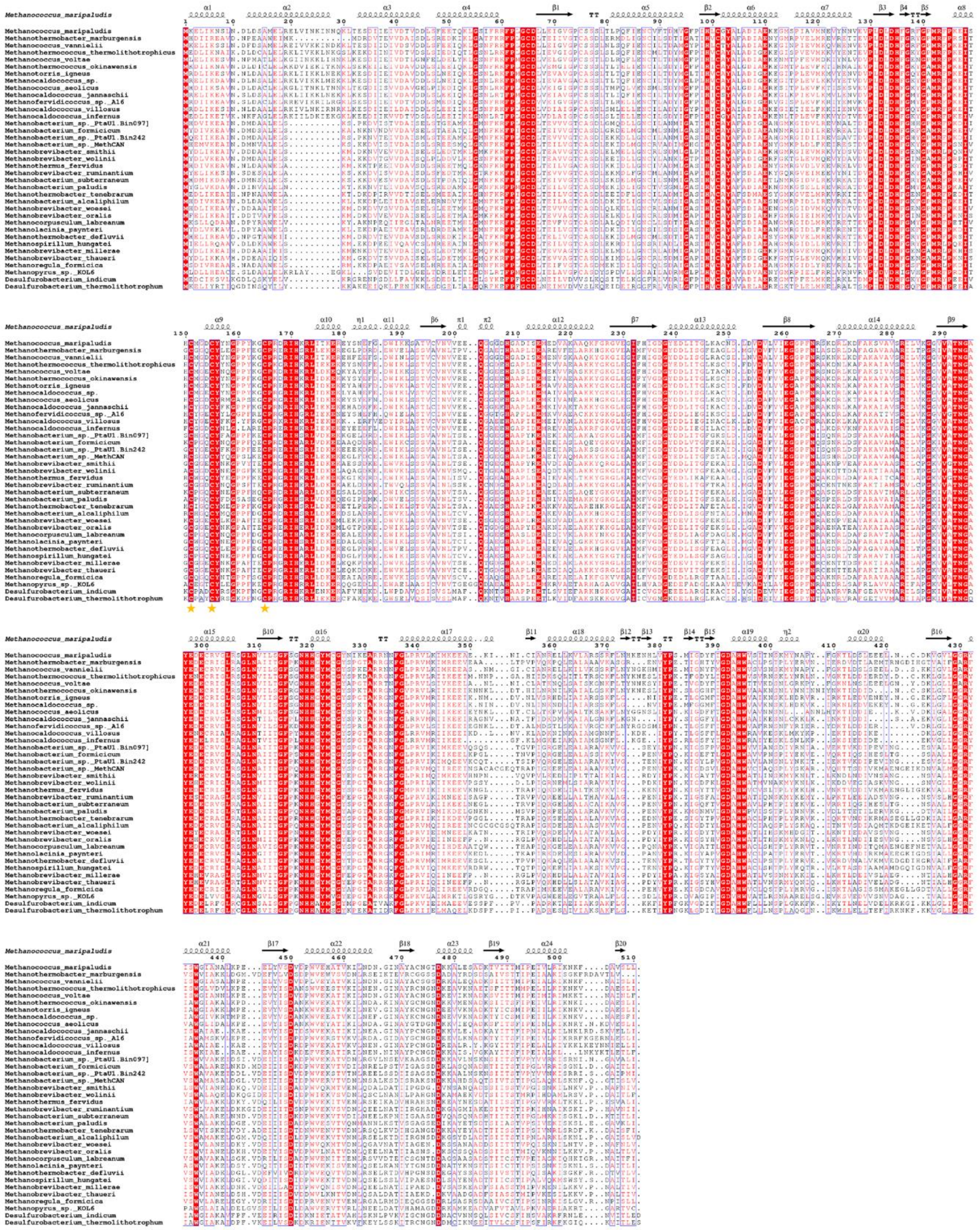

Figure S10. Multiple sequence alignments of HcgG from different microorganisms. The fully conserved amino-acid residues are shown in red. Potential cysteine residues responsible for the coordination of the possible [4Fe-4S]-binding site are highlighted by yellow stars under the sequences. The secondary structure elements were obtained from the AlphaFold model.

Table S1. Strains used in this work.

Genotype	Strain	Reference
Wild type *M. maripaludis*	S2	[9]
S2 Δ*upt*	Mm901	[10]
Mm901 Δ*frcfru*	Mm1280	[10]
Mm1280 Δ*hcgA*	Mm1328	[11]
Mm1280 Δ*hcgG*	Mm1333	[11]
Mm901+pWL40neo*HishcgG*	Mm2001	This work

Table S2. Proteome analysis of the Δhcg mutants.

		Peptide Spectrum Matches (PSM)			
Protein	Accession	*S2*	Δ*frh*	Δ*hcgA*	Δ*hcgG*
**FrhA**	WP_011171326.1	72	0	0	0
**Hmd**	WP_011170071.1	161	22	82	29
**HcgA**	WP_011170070.1	5	15	0	2
**HcgB**	WP_011171441.1	5	7	4	3
**HcgC**	WP_011171442.1	23	34	10	5
**HcgD**	WP_011169997.1	5	14	5	3
**HcgE**	WP_013999640.1	3	9	3	2
**HcgG**	WP_011170069.1	13	36	9	0

References

[1] M. Nakamura, K. Saeki, Y. Takahashi, *J. Biochem.* **1999**, *126*, 10-18.
[2] D. R. Fonseca, M. F. A. Halim, M. P. Holten, K. C. Costa, *J. Bacteriol.* **2020**, *202*.
[3] D. L. Tumbula, R. A. Makula, W. B. Whitman, *FEMS Microbiol. Lett.* **1994**, *121*, 309-314.
[4] K. C. Costa, P. M. Wong, T. Wang, T. J. Lie, J. A. Dodsworth, I. Swanson, J. A. Burn, M. Hackett, J. A. Leigh, *Proc. Natl. Acad. Sci. USA* **2010**, *107*, 11050-11055.
[5] F. Long, L. Wang, B. Lupa, W. B. Whitman, *Archaea* **2017**, *2017*, 7046026.
[6] S. Schaupp, F. J. Arriaza-Gallardo, H.-J. Pan, J. Kahnt, G. Angelidou, N. Paczia, K. Costa, X. Hu, S. Shima, *Angew. Chem. Int. Ed* **2022**, *61*, e202200994
[7] S. Shima, M. Schick, H. Tamura, *Methods Enzymol.* **2011**, *494*, 119-137.
[8] T. Watanabe, T. Wagner, G. F. Huang, J. Kahnt, K. Ataka, U. Ermler, S. Shima, *Angew. Chem. Int. Ed.* **2019**, *58*, 3506-3510.
[9] a) T. J. Lie, J. A. Leigh, *Appl. Environ. Microbiol.* **2007**, *73*, 6595-6600; b) W. B. Whitman, J. Shieh, S. Sohn, D. S. Caras, U. Premachandran, *Syst. Appl. Microbiol.* **1986**, *7*, 235-240.
[10] T. J. Lie, K. C. Costa, B. Lupa, S. Korpole, W. B. Whitman, J. A. Leigh, *Proc. Natl. Acad. Sci. USA* **2012**, *109*, 15473-15478.
[11] T. J. Lie, K. C. Costa, D. Pak, V. Sakesan, J. A. Leigh, *FEMS Microbiol. Lett.* **2013**, *343*, 156-160.

Author Contributions

S.Sh. directed the research. F.J.A.-G, S.Sc. and Y.-C.Z. performed sample preparation, *in vitro* biosynthesis and biochemical analysis. H.-J.P. and X.H. performed chemical synthesis. J.K performed proteome analysis. G.A. and N.P. performed HRes-LC-MS analysis. M.F.A-F. and C.K. constructed expression vector of HcgG. F.J.A.-G, S.Sc., Y.-C.Z. and S.Sh. analyzed the data. S.Sh. wrote the original draft and edited with contributions from other authors
